# Supplementary material for: Neurovascular Unit-Derived Extracellular Vesicles: From Their Physiopathological Roles to Their Clinical Applications in Acute Brain Injuries
Source: Biomedicines. 2022 Sep 1;10(9):2147. doi: 10.3390/biomedicines10092147 (PMC9495841; doi:10.3390/biomedicines10092147)
Supplement: Supplementary file 1 [file biomedicines-10-02147-s001.zip › Supplementary Table S1 -proofed.pdf]

Supplementary Table S1. Inclusion and exclusion criteria of studies reported in Table 1.

	Cohort	Inclusion criteria	Exclusion criteria	Reference
1.	AIS (*n* = 66) and TIA (*n* = 21) patients and healthy participants (*n* = 24)	For patients: (1) symptoms onset ≤ 48h; (2) age ≥18 years<br>Both: written informed consent		[132]
2.	AIS patients with LAA (*n* = 53) or SAO (*n* = 59) and healthy participants (*n* = 35)	For patients: (1) symptoms onset < 48h; (2) ischemic stroke confirmed by MRI<br>Both: written informed consent	For healthy participants: (1) infectious disease in the previous month; (2) history of autoimmune disorder or peripheral vascular disease; (3) TIA, cerebral infarction and cerebral hemorrhage; (4) liver and kidney failure; (5) cardiac dysfunction; (6) medication for lipid control, inflammation suppression or immunosuppression	[190]
3.	AIS patients (*n* = 44) and high-cardiovascular risk participants (*n* = 44)	For patients: (1) symptoms onset < 48h; (2) suspicion of ischemic stroke; (3) written informed consent<br>For controls: (1) high cardiovascular risk<br>Both: written informed consent	For controls: (1) no history of cardiovascular disease or cancer	[134]
4.	ICH patients (*n* = 36) and controls (*n* = 10)	For patients: (1) symptoms onset < 6h; (2) age > 40 and < 80 years; (3) CT-noted ICH; (4) no other previous systemic diseases including uremia, liver cirrhosis, malignancy, and chronic heart or lung disease (except diabetes mellitus and hypertension); (5) need and acceptance of surgical therapy; (6) time to surgery < 12 h<br>For healthy participants: (1) suspicion of SAH resulting in normal results on brain MRI scan; (2) no vascular risk factor (3) need and acceptance of lumbar puncture; (4) negative lumbar puncture<br>Both: written informed consent	For patients: (1) history of head trauma or previous stroke; (2) use of antiplatelet or anticoagulant medication; (3) history of arteriovenous malformation of the brain; (4) history of ruptured cerebral aneurysm	[130]
5.	SAO (*n* = 34), LAA (*n* = 41), cardioembolism (*n* = 20) and undetermined etiology (*n* = 15) patients and healthy participants (*n* = 61)	For patients: (1) acute-stage cerebral infraction at admission; (2) MRI and MRA within 6 h after symptoms onset	(1) Treatment with thrombolysis; (2) signs of early neurological resolution within 24 h; (3) failure to obtain informed consent; (4) urgent percutaneous transluminal angioplasty or intravascular surgery	[131]

6.	SAH patients (n = 20) and healthy participants (n = 20)	For patients: (1) symptoms onset < 48 h; (2) age >18 and <70 years; (3) SAH confirmed by CT, ruptured aneurysm demonstrated by angiography, endovascular coiling possible<br>Both: written informed consent	For patients: (1) moderate to severe CVS at screening; (2) known coagulopathies; (3) treatment with platelet aggregation inhibitors or vitamin K antagonists; (4) severe concomitant diseases	[128]
7.	AIS patients (n = 68) and healthy participants (n = 61)	For patients: (1) AIS patients with focal symptoms within 7 days from onset<br>Both: written informed consent	For patients: (1) history of inflammatory disease, renal, hepatic or hematological disorders; (2) history of autoimmune or malignant disease<br>For healthy participants: (1) history of stroke or chronic hepatic or renal disease	[133]
8.	SAH patients (n = 22) and healthy participants (n = 13)	For patients: (1) age >18 and < 80 years; (2) modified Fisher Grade 3 or 4; (3) a saccular aneurysm confirmed on cerebral angiography<br>Both: written informed consent	For patients: (1) symptoms onset >48h	[129]
9.	AIS patients (n = 41) and healthy participants (n = 23)	For patients: (1) AIS diagnosis<br>Both: written informed consent	For healthy participants: (1) older age; (2) no history of AIS	[136]

Abbreviations: AIS= acute ischemic stroke, IS = ischemic stroke, IPH= intraparenchymal hemorrhage, LAA= large artery atherosclerosis, MRA= magnetic resonance angiography, MRI= magnetic resonance imaging, SAH = subarachnoid hemorrhage, SAO = small artery occlusion, TIA= transient ischemic attack.
