# Supplementary material for: Neurovascular Unit-Derived Extracellular Vesicles: From Their Physiopathological Roles to Their Clinical Applications in Acute Brain Injuries
Source: Biomedicines. 2022 Sep 1;10(9):2147. doi: 10.3390/biomedicines10092147 (PMC9495841; doi:10.3390/biomedicines10092147)
Supplement: Supplementary file 1 [file biomedicines-10-02147-s001.zip › Supplementary Table S2 -proofed.pdf]

**Supplementary Table S2.** Inclusion and exclusion criteria of studies reported in Table 2.

| | Cohort | Inclusion criteria | Exclusion criteria | Reference |
|---|---|---|---|---|
| 1. | AIS patients (*n* = 65) and healthy participants (*n* = 66) | For patients: (1) MRI- or CT-confirmed IS<br>Both: written informed consent | For patients: (1) diagnosis of intracerebral hemorrhage or unknown etiology<br>For healthy participants: (1) no history of stroke | [192] |
| 2. | AIS patients (*n* = 50) and healthy participants (*n* = 50) | For patients: (1) MRI-confirmed IS<br>Both: written informed consent | For healthy participants: (1) no history of stroke | [193] |
| 3. | AIS patients (*n* = 50) | For patients: (1) symptoms onset <72 h<br>Both: written informed consent | For patients: (1) recurrent stroke; (2) renal or liver failure, acute infectious disease, tumor, hematologic disease; (3) patients who are unable to cooperate with physical examination<br>For healthy participants: (1) no history of stroke | [194] |
| 4. | hyperacute (*n* = 15), acute (*n* = 55), subacute (*n* = 31) and recovery phase (*n* = 32) IS patients and healthy participants (n = 24) | For patients: (1) recent onset of neurological symptoms, (2) CT- or MRI-confirmed IS diagnosis; (3) absence of other serious systemic complications | For patients: (1) diagnosis of cerebral haemorrhage; (2) history of tumor, or acute or chronic inflammation | [195] |
| 5. | IS (*n* = 21), IPH (*n* = 19) and SAH (*n* = 17) patients | (1) symptoms onset ≤24h; (2) written informed consent | | [196] |
| 6. | AIS patients (*n* = 38) and healthy participants | For patients: (1) ischemic stroke with a NIHSS score > 5, (2) symptoms onset <24 h<br>Both: (1) inclusion in the Oxford Vascular Study cohort | | [197] |

*Abbreviations: AIS= acute ischemic stroke, IS = ischemic stroke, IPH= intraparenchymal hemorrhage, LAA= large artery atherosclerosis, NIHSS=National Institutes of Health Stroke Scales, SAH = subarachnoid hemorrhage.*
