# Supplementary material for: Neurovascular Unit-Derived Extracellular Vesicles: From Their Physiopathological Roles to Their Clinical Applications in Acute Brain Injuries
Source: Biomedicines. 2022 Sep 1;10(9):2147. doi: 10.3390/biomedicines10092147 (PMC9495841; doi:10.3390/biomedicines10092147)
Supplement: Supplementary file 1 [file biomedicines-10-02147-s001.zip › Supplementary Table S3 -proofed.pdf]

Supplementary Table S3. Inclusion and exclusion criteria of studies reported in Table 3.

	Cohort	Inclusion criteria	Exclusion criteria	Reference
1.	mTBI patients (*n* = 47), healthy (*n* = 39) and orthopedically injured (*n* = 7) participants	For patients : (1) Glasgow Coma Scale score of 13–15; (2) high clinical suspicion of non-penetrating acute TBI; (3) age ≥18 years; (4) time of injury <24 h; (5) admission to the hospital; (6) written informed consent<br><br>For healthy and orthopedically injured participants: (1) no history of concussion/TBI	For all: (1) history of serious neurologic or psychiatric disease; (2) penetrating traumatic brain injury; (3) pregnancy; (4) incarceration	[210]
2.	TBI patients (*n* = 72) and healthy participants (*n* = 20)	For patients: (1) age ≥18 years; (2) ability to speak and read English; (3) diagnosis of non-penetrating TBI; (4) time of injury ≤1 year<br><br>For healthy participants: (1) age ≥18 years; (2) ability to speak and read English; (3) good medical and psychological health<br><br>Both: written informed consent	For patients: (1) pregnancy; (2) contraindication to MRI; (3) history of significant psychiatric or neurologic disease<br><br>For healthy participants: (1) history of alcohol or substance abuse; (2) history of congestive heart failure, cancer, or sleep disorders; (3) history of taking medicines that cause fatigue (beta blockers, diuretics, or narcotics); (4) no history of head injury	[211]
3.	mTBI military personnel (*n* = 42) vs. healthy participants (*n* = 22)	For patients: (1) diagnosis of mTBI due to a blow to the head and had a loss of consciousness of 20 min or less<br><br>For healthy participants: (1) no history of mTBI<br><br>Both: (1) deployment within the previous 18 months	Both: (1) history of drug or alcohol abuse; (2) a severe medical condition; (3) a severe psychiatric condition; (4) a severe neurologic;<br>(5) history of moderate-severe TBI	[212]
4.	Acute (*n* = 18) and chronic (*n* = 14) mTBI and healthy participants (*n* = 21)	For acute mTBI: (1) time of injury ≤7 days<br><br>For chronic mTBI: (1) at least 2 previous mTBI events; (2) time of injury >3 months	For healthy participants: (1) history of mTBI within the last 5 years	[213]
5.	Repetitive mTBI (*n* = 56), with 1-2 mTBI (*n* = 94) and without TBI (*n* = 45) participants	(1) suspicion of concussive event during combat deployment	(1) history of moderate-severe TBI with a Glasgow Coma Scale < 13 or coma duration > 0.5 h or post-traumatic amnesia duration > 24h, or CT-reported traumatic intracranial lesion; (2) history of major neurologic	[214]

				or psychiatric disorder (except PTSD and mood disorders)		
6.	Moderate–severe TBI patients (*n* = 21)	7.	(1) age ≥18 years; (2) diagnosis of non-penetrating moderate-severe TBI with Glasgow Coma Scale < 12	(1) pregnancy; (2) GCS of 3 with bilateral fixed and dilated pupils; (3) normal head CT; (4) neurological comorbidities that may impact biomarker concentrations	8.	[215]
9.	Former NFL players with CTE and repetitive TBI (*n* = 78) and participants with reported history of TBI (*n* = 15)		For NFL players: (1) male; (2) age > 40 and < 70; (3) participation in organized tackle football >12 years; (4) play in the NFL at positions with common extensive head impacts >2 years; (5) self-report of progressive cognitive, behavioral, and mood symptoms >6 months<br><br>For healthy participants: (1) male; (2) age >40 and <70; (3) participation in organized noncontact sports >4 years, with 2 years at the college level or beyond	For healthy participants: (1) history of military service; (2) participation in organized sports with expected high exposure to head impacts; (3) history of TBI;<br>(4) self-reported symptoms of cognitive, mood, or behavioral impairment <6 months		[216]
10.	With mTBI (*n* = 19) and without mTBI (*n* = 20) military personnel		(1) Participation in Marine Resilience Study-II			[217]

Abbreviations: CTE= Traumatic chronic encephalopathy, PTSD= post-traumatic stress disorder, NFL= National Football League.
